# The risk of malnutrition in patients with spinal cord injury during inpatient rehabilitation–A longitudinal cohort study

**DOI:** 10.3389/fnut.2023.1085638

**Published:** 2023-01-23

**Authors:** Irene Flury, Gabi Mueller, Claudio Perret

**Affiliations:** ^1^Nutritional Therapy Department, Swiss Paraplegic Centre, Nottwil, Switzerland; ^2^Clinical Trial Unit, Swiss Paraplegic Centre, Nottwil, Switzerland; ^3^Institute of Sports Medicine, Swiss Paraplegic Centre, Nottwil, Switzerland

**Keywords:** nutritional status, screening, paraplegia, quadriplegia, risk factor

## Abstract

**Background and aim:**

Patients with spinal cord injury (SCI) show an increased risk of malnutrition. Studies found that about 50% of patients with a recent SCI are affected by malnutrition when they enter a rehabilitation institution. However, there is a lack of data during the course and at discharge of initial rehabilitation as well as missing knowledge about the factors promoting such a risk. The aim of this study was to assess the risk of malnutrition in individuals with SCI 3 months post injury and at the end of inpatient rehabilitation and to identify factors associated with a high risk of malnutrition.

**Methods:**

Retrospective, monocentric, longitudinal cohort study, using the data set of the Swiss Spinal Cord Injury Cohort Study and additional data from the patients’ medical records. Individuals with SCI were assessed for the risk of malnutrition using the Spinal Nutrition Screening Tool 3 months post injury and at discharge from initial inpatient rehabilitation. Odds ratios (OR) for potential risk parameters were calculated.

**Results:**

Of the 252 participants included, 62% were at risk for malnutrition 3 months post injury and 40% at discharge (*p* = 0.000). Moderate to high risk of malnutrition was found regardless of age and BMI. The highest odds for an increased risk at 3 months post injury was identified in ventilator-dependent persons (OR 10.2). At discharge from inpatient rehabilitation, pressure injury (OR 16.3) was the most prominent risk factor.

**Conclusion:**

In the population with SCI the risk of malnutrition is widespread during inpatient rehabilitation, but also at discharge. Ventilated persons and persons with pressure injuries are clear risk groups and need special attention. Based on these findings and the known negative impact of malnutrition on clinical outcomes, the awareness of malnutrition should be increased in the population with SCI. Therefore, a regular and standardized screening of the malnutrition risk is highly recommended.

## 1. Introduction

Malnutrition is a known phenomenon after spinal cord injury (SCI), which can negatively affect clinical outcomes and lead to prolonged hospital stays (1, 2). Malnutrition during inpatient rehabilitation of individuals with SCI is therefore an important aspect, which needs special attention. A 52% longer rehabilitation time and a 9.2% higher 1-year mortality in malnourished persons with SCI was documented (1). Further, malnutrition was found to negatively affect functional recovery in patients with SCI (2). Thus, an optimal nutritional status should help optimize medical and neurological benefits and reduce the risk of nutrition-associated disadvantages such as increased susceptibility to infection and slower wound healing (3). It is reported that the prevalence for malnutrition at entry into a rehabilitative institution is as high as 50% in patients with SCI, depending on the criteria used to define malnutrition (2, 4). The pathophysiology of SCI and its consequences can affect the nutritional status (1). The cause of malnutrition is usually multifactorial. It includes increased nutrient utilization, decreased food intake and malabsorption (5). Comorbidities of SCI such as dysphagia or pressure injury could be reasons for a decreased nutrient intake or increased nutrient utilization. Poor nutritional status can be a consequence or cause of diverse comorbidities in SCI (6). In addition, in the aging population the reasons for SCI are mainly related to non-traumatic causes such as tumors, infections and other diseases (7), which have a strong impact on metabolism and are often associated with multimorbidity. This fact increases the susceptibility to malnutrition in this population.

Various personal and lesion dependent factors increase the risk of malnutrition in SCI patients at the beginning of first rehabilitation. The presence of additional comorbidities such as neurologic bowel dysfunction, infections, the presence of poor skin conditions/pressure injuries or loss of appetite etc. during the course of the first rehabilitation process further favor the appearance of malnutrition (1, 3). Therefore, nutritional status should not only be evaluated upon admission, but also re-evaluated repeatedly during the course of the stay, since above described factors and complications may change substantially during the course. In view of this, there is a lack of data of patients with a prolonged inpatient stay, as is the case during initial rehabilitation. Existing studies (2, 4) describe the risk for malnutrition at entry into a rehabilitative institution, but not thereafter. Therefore, the aim of this study was to assess the risk of malnutrition in individuals with SCI 3 months post injury and at discharge from inpatient rehabilitation (i.e., 5 to 9 months post injury). A secondary aim was to identify factors promoting a high risk for malnutrition in this population.

## 2. Materials and methods

### 2.1. Design and setting

This retrospective, monocentric, longitudinal cohort study evaluates data from patients of the Swiss Paraplegic Centre Nottwil, Switzerland and was approved by the local ethics committee. The study was conducted as a nested project of the Swiss Spinal Cord Injury Cohort Study (SwiSCI) (8), using the existing dataset of the SwiSCI study and additionally collected data from the clinical medical records of the selected patients. Participants were screened for the risk of malnutrition by means of the Spinal Nutrition Screening Tool (SNST) (9). Screening was performed 3 months after injury and at discharge from inpatient rehabilitation. The SNST risk score was used to assess the risk of malnutrition.

### 2.2. Study population

The study included men and women participating in the SwiSCI cohort study. They were 18 years or older and spent their initial inpatient rehabilitation at the Swiss Paraplegic Centre Nottwil due to SCI between 1 September 2011 and 31 August 2020. All participants agreed on using their medical records data for research purposes.

### 2.3. Assessment of malnutrition risk

The risk of malnutrition was assessed using the SNST, which is a SCI-specific and validated screening tool (9). It assesses several criteria, most of which are recognized predictors or symptoms of malnutrition, such as age, weight history and appetite. It further assesses SCI disease-specific factors that may influence nutritional status, such as level of SCI, skin condition, presence of comorbidities such as need for artificial ventilation, artificial nutrition, supplements and texture-modified diet, in addition to the ability to eat. Each criterion is rated with a score between 0 and 5. The total score reflects the level of malnutrition risk: SNST score/risk level: 0–10 = low, 11–15 = moderate, > 15 = high (9).

The risk of malnutrition was assessed at two different time points, 3 months post injury and at discharge. Most of the parameters came from the SwiSCI database, whereas the parameters appetite, nutrition form and a few missing anthropometric values were collected from the patients’ medical records.

At both time points, weight was measured to the nearest 100 gram using a calibrated floor scale. Patients were weighed for a total weight with clothes, shoes, and wheelchair. For body weight, the wheelchair with clothes and shoes on it was then weighed separately and subtracted from the total weight. The heights were self-reported values. For the calculation of the weight loss 3 months post injury, the weight 1 month (28 ± 12 days) after injury was taken into account; if this was not available, weight before injury was used. For the time point of discharge, the weight difference between 3 months post injury and discharge was calculated. Weight loss for determining the SNST score was defined as follows: Slight = weight loss < 5% of initial body weight, moderate = 5–10%, marked > 10% (10). Parameters such as age, gender, and height were recorded only once in SwiSCI, 1 month post injury. The same applies to chronic diseases such as diabetes or substance abuse. The additional parameters from the medical records were extracted for the two time points. Information was obtained from the nursing documentation and/or medical history of the nutrition and/or speech and language therapists. Oral nutritional supplements, enteral or parenteral nutrition were recorded by including the physician’s prescription and the control over the intake list. Data for the appetite and nutrition form as part of the SNST were not collected on a specific date due to a risk of bias, instead a 4 weeks back view in the medical records was taken, and the most common diet and portion size were considered. Thus, if someone consumed normal food on the recording date, but modified-texture diet the weeks before, modified- texture diet was recorded for calculation of the SNST. In addition to the parameters for the SNST, co-factors such as gender, severity of injury, cause of injury and nutritional counseling were collected.

### 2.4. Statistical analyses

For the statistical analyses, the data from the SwiSCI study and those from the manual extraction of medical records were merged in Excel (version 2019, Microsoft, WA, USA). From these data, the SNST sum score was calculated per patient and measurement time point. Subsequently, this data set was imported into PASW/SPSS (IBM, PASW 25, NY, USA) statistics software for further analyses. All data were checked for normal distribution by visual screening of box plots for each assessed parameter. The frequency and percentage of the total group of patients at each risk level for the 3 month and discharge time points were calculated. In addition, identical risk level subgroup analyses were completed. Potential predictors of malnutrition were: tracheal cannula, mechanical ventilation, pressure injury, pneumonia, BMI < 25, gender, age > 65, modified/artificial nutrition and nutritional care. These potential predictors were compared at both measurement time points using Man–Whitney-*U* tests. The overall longitudinal change of malnutrition risk (3 months post injury vs. discharge) was compared with a Wilcoxon Signed Rank test. The level of significance was set at a *p* ≤ 0.05. Additionally, Odds ratios (OR) for these potential predictors at 3 months post injury and at discharge were calculated. For the parameter BMI the subgroups were defined at BMI > 25 and BMI < 25, and for age at > 65 years and < 65 years.

## 3. Results

### 3.1. Overall analysis

**FIGURE 1:**
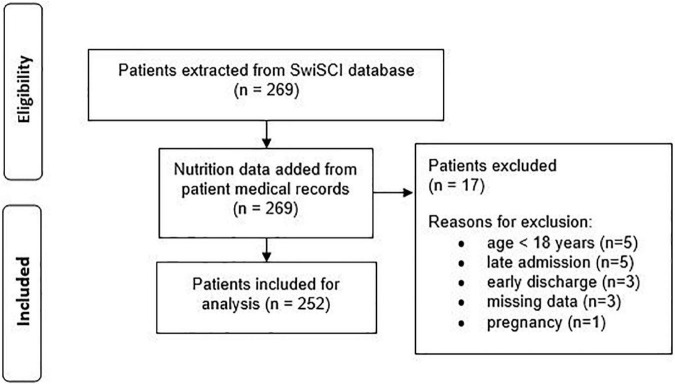
Patient inclusion flowchart.

**FIGURE 2:**
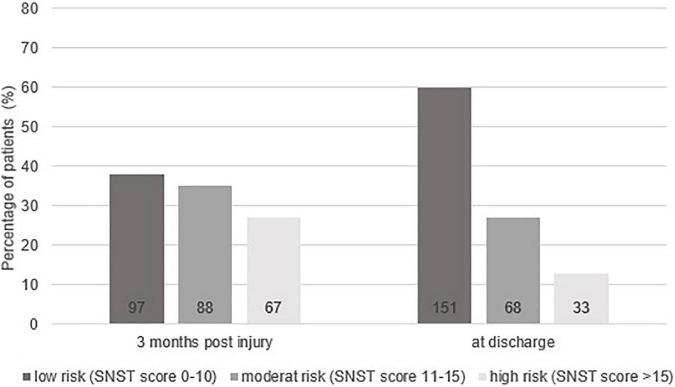
Overall percentage risk of malnutrition at 3 months post-injury and at discharge from inpatient rehabilitation for the three categories of the Spinal Nutrition Screening Tool (SNST). Absolute numbers of patients are shown at the bottom of the bars.

**FIGURE 3:**
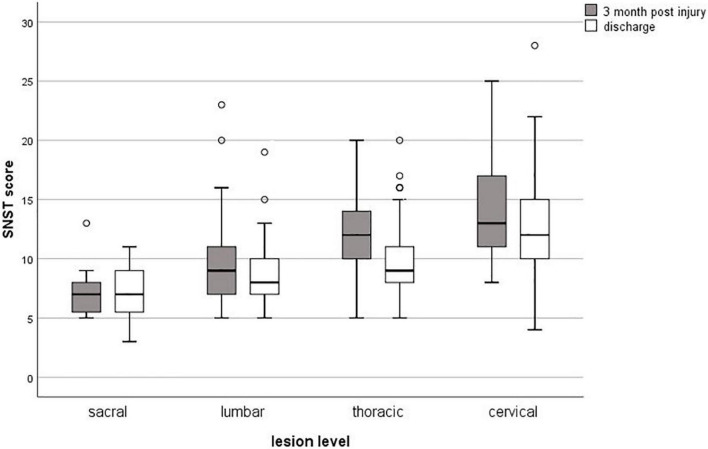
Box plots for the risk of malnutrition by level of SCI at 3 months post-injury and at discharge from inpatient rehabilitation. SNST, Spinal Nutrition Screening Tool.

The SwiSCI data set included 269 patients for the selected time-period. Figure 1 presents the patient eligibility criteria and lists reasons for their exclusion in the study. A total of 252 individuals were analyzed. Baseline characteristics of patients are presented in Table 1. Most of the data were not normally distributed, therefore, non-parametric data reporting and analyses have been chosen. Sixty-two percent of all participants had a moderate or high risk of malnutrition (SNST score > 10) 3 months post injury, which dropped significantly (*p* = 0.000) to 40% at discharge (Figure 2). The overall median SNST score 3 months post injury was 12 and 10 at discharge. Figure 3 shows the risk of malnutrition based on the level of SCI. The percentage of individuals with tetraplegia affected from a medium and especially from a high risk was higher compared to patients with paraplegia: 3 months post injury 43% of the individuals with tetraplegia were at high risk (SNSTscore > 15) vs. 17% of the persons with paraplegia. At discharge 26% of patients with a tetraplegia vs. 6% of patients with a paraplegia were at high risk of malnutrition.

**TABLE 1:** Characteristics of participants, n (%) or median interquartile range (IQR).

Characteristic	Overall	
*n*	252	
Age (years)	53 (40.0–63.5)	
Sex (male/female)	196/56 (78/22%)	
Cause of SCI (traumatic/non-traumatic)	173/79 (69/31%)	
Height (cm)	175 (170–181)	
Body weight at discharge (kg)	75 (66.3–84.4)	
BMI (kg/m^2^)	24.0 (21.3–26.9)	
**Level and severity of injury**	**3 months p. i. 86 days (77–94)**	**At discharge 193 days (152–259)**
Tetraplegia	94	86
Complete	15 (16%)	15 (17%)
Incomplete	79 (84%)	71 (83%)
Paraplegia	158	166
Complete	47 (30%)	47 (28%)
Incomplete	111 70%)	119 (72%)
AIS (A/B/C/D/E)	62/30/31/126/3 (25/12/12/50/1%)	62/21/25/135/9 (25/8/10/54/3%)
Level of SCI (C/T/L/S)	94/101/50/7 (37/40/20/3%)	86/101/50/15 (34/40/20/6%)

p. i., post injury. C, cervical; T, thoracic; L, lumbar; S, sacral; BMI, body mass index.

AIS, American Spinal Cord Injury Association Impairment Scale.

Note that in the course of rehabilitation 8 persons with tetraplegia became paraplegic.

The proportion of at risk patients 3 months post injury was 64% with complete (American Spinal Injury Association Impairment Scale/AIS A) (11) and 58% with incomplete (AIS B,C,D,E) SCI. Traumatic SCI showed a slightly higher percentage of patients at risk for malnutrition than non-traumatic SCI (66 vs. 59.5%). Additional absolute and relative numbers of patients at risk and with low risk for malnutrition at 3 months post injury and at discharge for subgroups are presented in Table 2.

**TABLE 2:** Malnutrition risk over the course of first rehabilitation overall and for different subgroups (absolute numbers and (%) reported for each parameter and timepoint).

	3 months post injury					At discharge				
	Overall	At risk		Low risk		Overall	At risk		Low risk	
	(*N*)	(N)	(%)	(N)	(%)	(N)	(N)	(%)	(N)	(%)
Number of participants	252	155	62%	97	38%	252	101	40%	151	60%
Mechanically ventilated	35	35	14%	0	0%	35	28	11%	7	3%
Non-ventilated	217	120	48%	97	38%	217	73	29%	144	57%
Presence of a tracheal cannula	11	11	4%	0	0%	3	3	1%	0	0%
No tracheal cannula	241	144	57%	97	39%	249	98	39%	151	60%
Pneumonia	47	44	17%	3	1%	0	n.a.		n.a.	
No pneumonia	205	111	45%	94	37%	252	101	40%	151	60%
Pressure ulcer (grade 1–5)	50	43	17%	7	3%	32	28	11%	4	2%
Intact skin conditions	202	112	44%	90	36%	220	73	29%	147	58%
BMI < 25 kg/m2	150	98	39%	52	20%	108	41	16%	67	27%
BMI ≥ 25 kg/m2	102	57	23%	45	18%	144	60	24%	84	33%
Male	196	125	50%	71	28%	196	83	33%	113	45%
Female	56	34	13%	22	9%	56	18	7%	38	15%
Age: >65 years	60	44	18%	16	6%	60	32	13%	28	11%
Age: ≤ 65 years	192	111	44%	81	32%	192	69	27%	123	49%
Modified/artificial nutrition	115	98	39%	17	7%	78	57	23%	21	8%
Normal nutrition	137	59	23%	78	31%	174	44	18%	130	51%
Nutritional care (nutritionist)	208	133	52%	75	30%	70	19	8%	51	20%
No nutritional care	44	22	9%	22	9%	182	82	32%	100	40%

SNST, Spinal Nutrition Screening Tool; BMI, body mass index. n.a., not applicable; At risk = SNST score ≥ 11, low risk = SNST score ≤ 10.

22 persons (9%) at risk for malnutrition did not receive nutritional therapy 3 months post injury. At discharge, 82 persons (33%) at risk for malnutrition had no nutritional therapy or had already completed it.

Malnutrition risk during rehabilitation decreased in 65 persons (26%) from 3 months post injury to discharge. It remained the same in 176 persons (70%). In 11 persons (4%), the risk of malnutrition increased from 3 months post injury until discharge.

### 3.2. Factors associated with malnutrition

Pressure injury and mechanical ventilation were nutrition-independent factors associated with the highest risk of malnutrition (Table 3). All ventilator-dependent persons (100%) had a high risk of malnutrition. Among those with pneumonia, 94% were at high risk and among those with pressure ulcers 86%. Nutrition dependent factors associated with a high risk of malnutrition were a modified and/or artificial nutrition and the inability to eat independently.

**TABLE 3:** Median SNST score for subgroups; *p*-values and odds ratios (OR) for differences between the respective subgroups.

		3 months post injury			At discharge		
		SNST score median (IQR)	*P*-value	OR (95% CI)	SNST score median (IQR)	*P*-value	OR (95% CI)
Cause of SCI	Traumatic	11.0 (9–15)			10.0 (8–12)		
	Non-traumatic	12.0 (10–15)	0.545	0.9 (0.5–1.6)	10.0 (8–12)	0.435	1.4 (0.5– 3.7)
Age	>65 years	13.5 (10.0–16.5)			11.0 (9–14)		
	≤65 years	11.0 (9–14)	0.001*	2.7 (1.4–5.3)	10.0 (8–11.5)	0.000*	1.9 (0.8–4.6)
Sex	Male	12.0 (9.0–15)			10.0 (8–12)		
	Female	11.0 (9–14)	0.160	1.8 (0.8–4.1)	9.0 (8–12)	0.565	1.5 (0.5–4.5)
BMI	≥25 kg/m2	12.0 (10–15)			10.0 (8–13)		
	<25 kg/m2	11.5 (9–15)	0.283	1.1 (0.6–2.0)	10.0 (8–14)	0.112	0.2 (0.1–0.7)
Tracheostomy	No	12.0 (10–14.5)			10.0 (8–12)		
	Yes	17.0 (13–22)	0.000*	8.6 (3.4–21.8)	18.0 (18–23)	0.004*	n.a.
Ventilatory support	No	11.0 (9–14)			9.0 (8–11)		
	Yes	17.0 (14–20)	0.000*	10.2 (4.6–22.2)	14.0 (13–17)	0.000*	5.8 (2.3–14.4)
Pneumonia	No	12.0 (10–15)			10.0 (8–12)	n.a.	n.a.
	Yes	14.0 (11–17)	0.028*	7.2 (3.6–14.4)	n.a.	n.a	n.a.
Pressure injury (grade 1–5)	No	11.0 (9–14)			10.0 (8–11)		
	Yes	14.0 (12–20)	0.000*	4.3 (2.2–8.5)	15.0 (12.0–18.5)	0.000*	16.3 (6.4–42.0)
Nutrition form	Normal nutrition	10.0 (9–12)			9.0 (8–10.5)		
	Modified/artificial	15.0 (12–17)	0.000*	18.9 (7.2–49.7)	12.0 (10–15)	0.000*	10.9 (3.9–30.5)
Meal intake	Independently	12 (10–14)			10.0 (8–11)		
	Need to be fed	16.5 (13–20)	0.000*	6.8 (2.8–16.4)	16.0 (13–19)	0.000*	23.2 (8.6–62.1)

SNST, Spinal Nutrition Screening Tool; BMI, body mass index;

*, Significant difference; IQR, interquartile range; CI, confidence interval; n.a., not applicable.

## 4. Discussion

The main finding of the present study was a widespread risk of malnutrition, affecting over 60% of individuals with SCI during inpatient rehabilitation. This risk was higher and more common in persons with tetraplegia compared to paraplegia. Our findings are consistent with the existing literature at entry into initial rehabilitation (2, 4) and add data on nutritional status of patients 3 months post injury and at discharge from inpatient rehabilitation. Length of stay (LOS) for initial inpatient rehabilitation in Switzerland after SCI is usually several months (8). In most European countries this is similar (12–15). The medical condition, nutrient requirements and other specific influencing factors can change during this time. Nutrition independent factors associated whit a high risk of malnutrition are pressure injury and ventilation dependency.

### 4.1. Age

There was a significant and 2-3-fold higher malnutrition risk in individuals aged > 65 years compared to those aged ≤ 65 years. It is known from non-SCI specific literature (16) that malnutrition is most prevalent in geriatric patients and that age is a predisposing factor for nutritional deficiencies in hospitalized individuals. The ESPEN (European Society for Clinical Nutrition and Metabolism) guidelines on clinical nutrition and hydration in geriatrics describes the elderly as a vulnerable person in relation to malnutrition and suggests strategies for the early detection and treatment of malnutrition (16). The German hospital malnutrition study showed the highest prevalence (56%) of malnutrition in geriatric patients. In a multivariate analysis, age was found to be one of the strongest influencing factors for the development of malnutrition (17) supporting the result of the present study identifying age as a risk of malnutrition in individuals with SCI.

### 4.2. Cause of SCI

The proportion of non-traumatic injuries in the SCI population has increased over the last decades and has surpassed the proportion of traumatic injuries (7, 18). This might be explained by the increasing life expectancy of people with SCI. A non-traumatic SCI often results from age-associated diseases such as tumors, circulatory disorders, and abscessing infections (7). Because these conditions, particularly tumors, infections, and the age factor are additionally associated with high-risk potential for malnutrition, a higher proportion of patients at risk for malnutrition was expected in this study for the non-traumatic cause. The results are in contrast to this expectation. There was no significant difference (*p* = 0.545/OR 0.9) in the median score of malnutrition risk between traumatic and non-traumatic SCI, and only a slightly higher percentage of individuals with SCI were at risk for malnutrition in the traumatic group (66 vs. 59.5%).

### 4.3. BMI

In terms of BMI, our results clearly reflect the clinical experience concerning the population with SCI. The classic visual image of a malnourished patient as a thin, lean appearance is still prevalent. However, malnutrition is not exclusive to lean persons, as demonstrated by this study. In total, 40% of the included participants were overweight (BMI ≥ 25) and showed an increased median risk for malnutrition at 3 months post injury (SNST score of 12). Wong et al. (4) also found a risk of malnutrition in 45% of the overweight patients (BMI > 25). The group of normal and overweight persons should not be underestimated in terms of malnutrition because of their appearance and BMI. The phenomenon of sarcopenic obesity (19) may be widespread in the population with SCI due to the change in body composition after an injury of the spinal cord. During the course of initial rehabilitation after SCI, energy consumption decreases (20), whereas overweight and obesity are widespread in this population (21). In view of the low lean body mass, usually increased fat mass and subsequent metabolic changes, a BMI cut-off point of 22 for overweight and 27 for obesity is recommended (22) for the SCI population. Consequently, when using these cut-off values, significantly more individuals with overweight would be at risk of malnutrition in the present study.

### 4.4. Factors associated with a high risk of malnutrition

Comorbidities associated with the highest risk of malnutrition were identified as: pressure injuries, pneumonia and ventilation dependency.

#### 4.4.1. Artificial ventilation

Individuals on artificial ventilation have an about 10 times higher risk (OR 10.2) of malnutrition at 3 months post injury compared to individuals without artificial ventilation. Wong et al. (4) also found a high risk of malnutrition in the mechanically ventilated group, particularly those with a tracheostomy, whereas 56.3% of mechanically ventilated vs. 38.7% of non-ventilated patients were affected by malnutrition. In this group, most had tetraplegia, which accumulates the risk factors for malnutrition such as dysphagia or ventilator-dependency (4). In the study of Pelekhaty et al. (23) with ventilator-dependent SCI patients in the acute phase, the difficulty of avoiding hyper- as well as hypoalimentation was mentioned. This is because common nutritional estimation formulas for ventilator-dependent patients with SCI are not validated and may therefore be inaccurate. Due to the fact that hyper- and hypoalimentation negatively affect clinical outcomes and may prolong ventilation time (23), it makes them a particularly sensitive group in terms of malnutrition. Adequate nutritional support is therefore crucial for the care of these patients.

#### 4.4.2. Pneumonia

Individuals with pneumonia have an about 7 times higher risk (OR 7.2) compared to individuals without pneumonia. An increased energy requirement is evident in inflammatory events of any kind (24), making pneumonia a risk factor for malnutrition. The present study showed an increased risk for malnutrition in patients with pneumonia. It is evident that several interactions may play a role, such as the ventilatory situation due to pneumonia, or dysphagia which is common in cervical SCI as a cause for pneumonia (25).

#### 4.4.3. Pressure injury

The risk of malnutrition in the group with pressure injury was significantly higher 3 months post injury and at discharge (OR 4.3/OR 16.3, *p* = 0.000) than in the patients with intact skin conditions. Of those with pressure injury, 86% were at risk of malnutrition. At the time of discharge, overall fewer patients were affected by a pressure injury (*n* = 32), but 87% of these discharged presented with an increased risk of malnutrition. In the study by Wong et al. (4) 53% of the patients with pressure injury were affected by malnutrition. There is high evidence for a strong association between malnutrition and wound healing in the non-SCI specific literature (26). This is due to an increasing need for energy, protein and micronutrients associated with the wound situation (26). In addition, a high-grade pressure injury is usually associated with weeks of bed rest and a regression in rehabilitation, which can negatively affect mood, appetite and eating ability (27). This vicious circle is often observed in clinical routine. Therefore, nutritional therapy support is essential for these patients.

### 4.5. Nutrition associated factors

#### 4.5.1. Modified/artificial nutrition

Dysphagia is a common comorbidity of cervical SCI. As an intervention for swallowing difficulties a texture-modified diet (TDM) is often necessary. A recent review showed a compromised nutritional status and poor mealtime satisfaction in consumers of TMD. Therefore a TMD correlated with weight loss or malnutrition (28). This knowledge supports the result of this study that SCI patients with a TDM have a significantly higher risk of malnutrition than those with a normal nutrition (*p* = 0.000; OR 18.9). Artificial Ventilation and nutrition are often necessary in the initial phase. The energy expenditure is individual and difficult to estimate in this population, therefore indirect calorimetry is recommended as gold standard for individual determination (23). Farkas et al. (29) summarize in their review, that individuals with acute SCI do not demonstrate a hypermetabolism compared to other trauma conditions. Nevertheless, they appear to be in a negative energy balance with a total daily energy expenditure of 2030 to 3344 kilocalories (kcal) versus an energetic intake of 755–2290 kcal during the acute phase. An appropriate consumption or feeding of nutrients in the acute phase can therefore be a challenge. Especially during oralization, when artificial nutrition is needed in combination with TMD, there is a risk of an unadapted nutrition intake. Despite the significant difference to normal nutrition, artificial nutrition cannot be interpreted as a risk factor for malnutrition, because it also represents the therapy for a malnutrition that may already exist.

#### 4.5.2. Dependency on food intake support

People who need to be fed have a significantly higher risk of malnutrition compared to people who can eat independently (*p* = 0.000; OR 6.8). It is conceivable that people who are suddenly in need of mealtime assistance due to SCI might eat less or be under pressure when eating. However, this has not been adequately investigated and there may as well exist an interaction of different factors. People who are dependent on food intake during initial rehabilitation, are often those with cervical SCI and/or higher age. These factors are also associated with an increased risk of malnutrition.

### 4.6. Strengths and limitations

The strengths of this study are the high number of participants, the longitudinal design and the homogeneous group that included first rehabilitation patients only. This study is the first to present data related to malnutrition at discharge from initial rehabilitation.

A general limitation is that the terminology around malnutrition is used very differently in the literature. The exact definition of weight loss was missing in the SNST, so the definition of the Malnutrition Universal Screening Tool (MUST) (10) was used. The SNST can only be used to calculate the risk for malnutrition; it cannot be used for diagnostics of malnutrition itself. Further, no statement can be made about the interaction of the influencing factors as these were not investigated (e.g., how age may influence comorbidities and therefore malnutrition). Finally, the study population does not reflect the general population with SCI due to the very high proportion of traumatic injuries and the lower median age, which may be associated with traumatic injuries.

## 5. Conclusion

The present study showed a high and widespread risk of malnutrition in individuals with SCI during initial rehabilitation and also at discharge. Due to the known negative impact of malnutrition on the clinical outcomes of individuals with SCI, awareness of malnutrition needs to be raised, with a focus on improving procedures to identify these patients. A regular and standardized screening of the malnutrition risk is highly recommended and adequate nutritional support should be initiated. Special attention must be given to patients dependent on artificial ventilation, with fragile skin conditions, pneumonia, and those who cannot eat independently. For future research, knowledge about the interactions of influencing factors is needed. Malnutrition in chronic SCI and in other groups with progredient neurodegenerative diseases (e.g., multiple sclerosis, amyotrophic lateral sclerosis) should also be investigated.

## Data availability statement

The original contributions presented in this study are included in the article/supplementary material, further inquiries can be directed to the corresponding author.

## Ethics statement

The studies involving human participants were reviewed and approved by Ethikkommission Nordwest- und Zentralschweiz, Basel. The patients/participants provided their written informed consent to participate in this study.

## Author contributions

All authors contributed to study design, data analysis, supervision, and manuscript editing and approved the submitted version.
